# Self-supervised learning for using overhead imagery as maps in outdoor range sensor localization

**DOI:** 10.1177/02783649211045736

**Published:** 2021-09-28

**Authors:** Tim Y. Tang, Daniele De Martini, Shangzhe Wu, Paul Newman

**Affiliations:** 1 Mobile Robotics Group, University of Oxford, Oxford, UK; 2 Visual Geometry Group, University of Oxford, Oxford, UK

**Keywords:** Localization, cross-modality localization, deep learning, self-supervised learning

## Abstract

Traditional approaches to outdoor vehicle localization assume a reliable, prior map is available, typically built using the same sensor suite as the on-board sensors used during localization. This work makes a different assumption. It assumes that an overhead image of the workspace is available and utilizes that as a map for use for range-based sensor localization by a vehicle. Here, range-based sensors are radars and lidars. Our motivation is simple, off-the-shelf, publicly available overhead imagery such as Google satellite images can be a ubiquitous, cheap, and powerful tool for vehicle localization when a usable prior sensor map is unavailable, inconvenient, or expensive. The challenge to be addressed is that overhead images are clearly not directly comparable to data from ground range sensors because of their starkly different modalities. We present a learned metric localization method that not only handles the modality difference, but is also cheap to train, learning in a self-supervised fashion without requiring metrically accurate ground truth. By evaluating across multiple real-world datasets, we demonstrate the robustness and versatility of our method for various sensor configurations in cross-modality localization, achieving localization errors on-par with a prior supervised approach while requiring no pixel-wise aligned ground truth for supervision at training. We pay particular attention to the use of millimeter-wave radar, which, owing to its complex interaction with the scene and its immunity to weather and lighting conditions, makes for a compelling and valuable use case.

## 1. Introduction

The ability to localize relative to an operating environment is central to robot autonomy. Localization using range sensors, such as lidars (Levinson and Thrun, 2010; Wolcott and Eustice, 2015) and, more recently, scanning millimeter-wave radars (De Martini et al., 2020; Park et al., 2019; Saftescu et al., 2020), is an established proposition. Both are immune to changing lighting conditions and directly measure scale, while the latter adds resilience to weather conditions.

Current approaches to robot localization typically rely on a prior map built using a sensor configuration that will also be equipped on-board, for example a laser map for laser-based localization. This article looks at an alternative method. Public overhead imagery such as satellite images can be a reliable map source, as they are readily available, and often capture information also observable, albeit perhaps in some complex or incomplete way, by sensors on the ground. We can pose the localization problem in a natural way: finding the pixel location of a sensor in an overhead (satellite) image given range data taken from the ground. The task is, however, non-trivial because of the drastic modality difference between satellite images and sparse, ground-based radar or lidar.

Recent work on learning to localize a ground scanning radar against satellite images by Tang et al. (2020b) provides a promising direction which addresses the modality difference by first generating a synthetic radar image from a satellite image. The synthetic image can then be “compared” against live radar data, expressed as 2D images from a “bird’s eye” perspective, for pose estimation. Such an approach learns metric, cross-modality localization in an end-to-end fashion, and therefore does not require hand-crafted features limited to a specific environment.

The method in Tang et al. (2020b) trains a multi-stage network, and needs pixel-wise aligned radar and satellite image pairs for supervision at all stages. This, in turn, relies on sub-meter and sub-degree accurate ground-truth position and heading signals, which in practice requires high-end GPS/inertial navigation system (INS) and possibly bundle adjustment along with other on-board sensor solutions, bringing in burdens in terms of cost and time consumption.

To address this issue, building on the work of Tang et al. (2020b), we propose a method for localizing against satellite imagery that is learned in a self-supervised fashion. The core idea is still to generate a synthetic image with the appearance and observed scenes of a live range sensor image, but pixel-wise aligned with the satellite image. Yet, we relax the requirement on pixel-wise aligned data pairs and assume only a coarse initial pose estimate is available from a place recognition system, such that there is reasonable overlap between the live ground sensor field of view and a queried satellite image. Our method does not solve the global localization problem. Instead, given a coarse initial pose estimate from place recognition, our method solves the metric localization of a range sensor using overhead imagery, providing a refined SE(2) metric pose parametrized as ${\left[\begin{array}{ccc} x & y & \theta \end{array}\right]}^{T}.$

**Fig 1.**
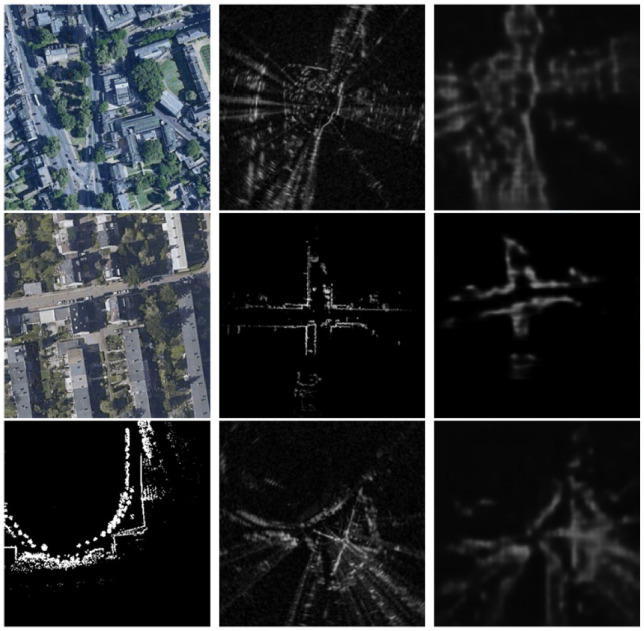
Given a map image of modality A (left) and a live data image of modality B (middle), we wish to find the unknown SE(2) offset between them. To do so, our method generates a synthetic image of modality B (right) that is pixel-wise aligned with the map image, but contains the same appearance and observed scenes as the live data image. Top: localizing radar data against satellite imagery. Middle: localizing lidar data against satellite imagery. Bottom: localizing radar data against prior lidar map.

Vitally, here we make no use of metrically accurate ground truth for training. Note also that although designed for localizing against satellite imagery, our method can naturally handle other forms of cross-modality registration, such as localizing a radar against a prior lidar map. Figure 1 shows synthetic images generated by our method used for pose estimation.

To the best of the authors’ knowledge, our proposed method is the first to learn the cross-modality, metric localization of a range sensor in a self-supervised fashion. Our method is validated experimentally on multiple datasets and achieves performances on-par with a state-of-the-art, supervised approach. Even though our method does not solve the global localization problem and instead relies on an external place recognition system, it can nevertheless be utilized to greatly refine the sensor’s metric pose starting from a coarse initial pose estimate, all in the absence of any prior sensor maps.

This article is an extended version of our prior work (Tang et al., 2020a). The improvements include a more detailed explanation for the motivation of our method to tackle the problem of localizing range sensors using satellite imagery (Section 3) and a more thorough description of our method (Section 4). For experimental validation (Section 6), we present additional qualitative results, an ablation study with reduced training data, a study on the trade-offs between network width and depth and solution quality, and an analysis on the choice of image resolution. We also introduce an introspective strategy at inference time to handle initial pose offsets larger than in the training data. Finally, we show with unsupervised domain adaptation, models trained using radar data can be utilized for localization between lidar and overhead imagery, and vice versa.

## 2. Related work

Our approach is related not only to other works in the field of localization using overhead imagery and the general theme of cross-modality localization, but also to learned methods for range sensor state estimation and unsupervised image generation. We provide a broad coverage of the most relevant research in these subjects in this section.

### 2.1. Localization using overhead images

Localization using aerial or overhead images has been of interest for the community for over a decade. The methods in Leung et al. (2008), Li et al. (2014), and Parsley and Julier (2010) localize a ground camera using aerial images, by detecting Canny edges from aerial imagery, and matching against lines detected by a ground camera. Several other vision-based approaches project the ground camera images to a top-down perspective via a homography, and compare against the aerial imagery by detecting lane markings (Pink, 2008), Speeded Up Robust Features (SURF) (Noda et al., 2010), or dense matching (Senlet and Elgammal, 2011). Recent work by Chebrolu et al. (2019) localizes a ground robot in a crop field by matching camera features against landmarks from an aerial map, and explicitly incorporates semantics of crops to reduce ambiguity.

Metric localization of range sensors or point-clouds against overhead imagery requires further pre-processing owing to the modality difference. Kaminsky et al. (2009) projected point-clouds into images and matched against binary edge images from overhead imagery. The method of Kaminsky et al. (2009) also constructs a ray image by ray-tracing each point, and introduces a free-space cost to aid the image registration. The work by de Paula Veronese et al. (2015) accumulates several lidar scans to produce dense lidar intensity images, which are then matched against satellite images utilizing normalized mutual information. Similar to Kaminsky et al. (2009), several other methods also pre-process the aerial image before matching against ground laser observations, for example using edge detection (Kümmerle et al., 2011) or semantic segmentation (Dogruer et al., 2010). In contrast to these approaches, our method directly learns the metric localization of a range sensor end-to-end, without the need for careful pre-processing or manual feature definition.

Closely related to our method is the seminal work on learning to localize a ground radar against satellite imagery by Tang et al. (2020b). As discussed previously, the method in Tang et al. (2020b) requires pixel-wise aligned ground truth for supervision, whereas our method is self-supervised.

### 2.2. Cross-modality localization

Other forms of cross-modality localization have also been heavily studied by the community. Several works propose to localize a forward-facing camera against a prior 3D point-cloud map (Caselitz et al., 2016;Wolcott and Eustice, 2014; Xu et al., 2017). Carle and Barfoot (2010) localized a ground laser scanner against an orbital elevation map. The works in Wang et al. (2019), Boniardi et al. (2017), Wang et al. (2017), and Mielle et al. (2019) localize an indoor lidar or stereo camera against architectural floor plans. Recently, Yin et al. (2021) proposed a joint learning system for radar place recognition using a prior lidar database, and achieves state-of-the-art results on the Oxford Radar RobotCar Dataset (Barnes et al., 2020) and MulRan Dataset (Kim et al., 2020).

OpenStreetMap is a particularly useful publicly available resource for robot localization. Brubaker et al. (2013) and Floros et al. (2013) concurrently proposed matching visual odometry paths to road layouts from OpenStreetMap for localization. Ruchti et al. (2015) proposed a road classification scheme to localize a ground lidar using OpenStreetMap. Yan et al. (2019) utilized networks pre-trained for point-cloud semantic segmentation, and built a light-weight descriptor to recognize intersections and gaps, and compared against the descriptors of the operating environment built using OpenStreetMap.

### 2.3. Learning-based state estimation for range sensors

A number of recent works were proposed for learning the odometry or localization of lidars. Barsan et al. (2018) represented lidar data as intensity images, and learned a deep embedding specifically for metric localization that can be used for direct comparison of live lidar data against a previously-built lidar map. Other methods such as Cho et al. (2019); Li et al. (2019), instead, learn deep lidar odometry by projecting lidar point-clouds into other representations before passing through the network. Lu et al. (2019b) learned descriptors from input point-clouds, and utilized 3D CNNs for solving SE(2) metric localization by searching in a 3D cost volume. In their later work, Lu et al. (2019a) proposed a method to learn SE(3) lidar point-cloud registration end-to-end. Recently, OverlapNet (Chen et al., 2020) was proposed to learn lidar loop-closure detection based on the overlap between bird’s eye view lidar images.

As an emerging sensor for outdoor state estimation, learning-based methods were proposed for scanning frequency-modulated continuous-wave (FMCW) radars. Aldera et al. (2019) utilized an encoder–decoder on polar image representation of radar scans to reject superfluous points for decreasing computation time in the classical radar odometry method described by Cen and Newman (2019). Barnes et al. (2019) learned image-based radar odometry by masking out regions distracting for pose estimation. Barnes and Posner (2020) learned point-based radar odometry by detecting key points from radar images. Saftescu et al. (2020) encoded images of polar radar scans through a rotation-invariant architecture to perform topological localization (place recognition), which can then be used for querying a previously built map (De Martini et al., 2020). These methods, however, are designed to compare data of the same sensor type, and do not address modality difference. Our approach is similar to Barsan et al. (2018), Cho et al. (2019), Aldera et al. (2019), Barnes et al. (2019), Weston et al. (2019), Saftescu et al. (2020), Barnes and Posner (2020), and Broome et al. (2020) in that we also represent range sensor data as 2D images.

### 2.4. Unsupervised image generation

A fundamental step in our approach is the generation of a synthetic image before pose computation, where there is no pixel-wise aligned target image for supervision. CycleGAN (Zhu et al., 2017) achieves unsupervised image-to-image transfer between two domains X and Y, by learning two pairs of generators and discriminators, and enforcing cycle-consistency when an image is mapped from X to Y and back from Y to X, and vice versa. Other methods (Lee et al., 2018; Liu et al., 2017) also utilize cycle-consistency but make different assumptions on how the latent spaces of the two domains are treated. Whereas these methods are concerned with generating photo-realistic images, we are interested in the problem of metric localization. As such, we need to explicitly encourage the synthetic image to contain information appropriate for pose estimation, rather than aiming for photo-realistic reconstruction.

Several prior works are also geometry-aware. The methods by Shu et al. (2018), Wu et al. (2019), and Xing et al. (2019) use separate encoders and/or decoders to disentangle geometry and appearance. The results are networks that can separately interpolate the geometry and appearance of the output images. Similarly, our method separately encodes information about the appearance and the relative pose offset, resulting in an architecture where the two are explicitly disentangled.

## 3. Overview and motivation

We seek to solve for the SE(2) pose between a map image of modality A and a live data image of modality B. Our main focus is when modality A is satellite imagery, whereas modality B are range sensor data represented as an image.

Previously, Radar–Satellite Localization Network (RSL-Net) (Tang et al., 2020b) was proposed to solve for the metric localization between matched pairs of radar and satellite images. In particular, it aims to generate a synthetic image that preserves the appearance and observed scenes of the live radar image, and is pixel-wise aligned with the paired satellite image. The synthetic image and the live radar image are then projected onto deep embeddings, where their pose offset is found by maximizing a correlation surface. We follow the same general approach, but, unlike RSL-Net, our method learns in a self-supervised fashion.

### 3.1. Hand-crafting features versus learning

Some of the works listed in Sections 2.1 and 2.2 can achieve decent accuracy on localizing a ground range sensor against aerial imagery. However, they typically rely on pre-processing the aerial images using hand-crafted features or transformations designed for a specific set-up and may not generalize to other sensors or different environments. For example, Kümmerle et al. (2011) focus on detecting edges from a campus dominated by buildings. de Paula Veronese et al. (2015) directly match accumulated lidar intensity images against aerial imagery without pre-processing, yet this is inappropriate for radars due to the complexity of their return signals.

**Fig. 2.**
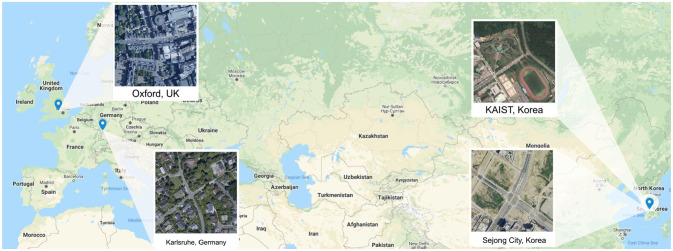
Our method is demonstrated on datasets collected around different locations, at various types of settings including urban (Oxford, UK), residential (Karlsruhe, Germany), campus (KAIST, Korea), and highway (Sejong City, Korea).

Our data-driven approach instead learns to directly infer the geometric relationship across modalities, remaining free of hand-crafted features. We show in Section 6 the robustness of our method when localizing against satellite imagery in various types of scenes, including urban, residential, campus, and highway (Figure 2).

### 3.2. Generating images versus direct regression

A naive approach would be to take a satellite image and a live data image as inputs, and directly regress the pose. Yet, as shown in Tang et al. (2020b), this led to poor results even for the supervised case. Our hypothesis is that when the two images are starkly different in appearance and observed scenes, the problem becomes too complex for direct regression to succeed given current techniques.

Generating synthetic images prior to pose estimation brings two advantages over directly regressing the pose. First, it is a simpler and less ill-posed problem than directly regressing the pose, particularly because we can utilize the live data image to condition the generation. Second, the image generation loss is distributed over an entire image of H×W pixels, where H and W are height and width, instead of on just three pose parameters that describe an SE(2) displacement ( x,y, and θ ), introducing greater constraints during optimization.

### 3.3. Conditional image generation

We tackle the synthesis of an image of the live data modality B from one of the map modality A as a conditional image generation task, that is, taking both a map (e.g., satellite) image and a live data image as inputs. An alternative approach is to learn a domain adaptation directly from A to B, without conditioning on the live data image, for example, standard image-to-image transfer such as CycleGAN (Zhu et al., 2017).

**Fig. 3.**
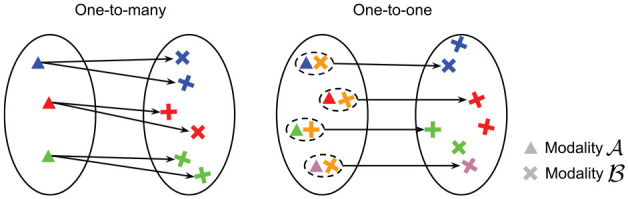
A one-to-many mapping (left) versus a one-to-one mapping (right). Left: the mapping from modality A to modality B preserves color, but is ambiguous in orientation of the output, resulting in a one-to-many mapping, and is therefore not a function. Right: augmenting the input with an element of B offers additional constraint in orientation, resulting in a one-to-one mapping as the mapping is now unambiguous in both color and orientation. Note that the mapping on the right is one-to-one, but not necessarily *surjective*.

**Fig. 4.**
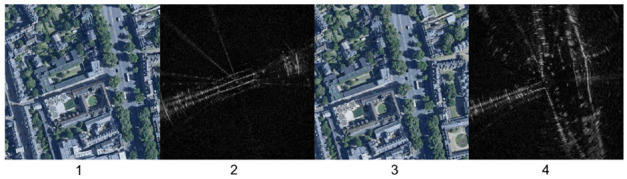
Two radar images captured 15 seconds apart from each other (2 and 4), pixel-wise aligned with satellite images (1 and 3). Though the overlapping scenes in the satellite images are identical, the radar scans appear significantly different, as they capture different regions in their field of view.

In practice, the map (e.g., satellite) image is a denser representation of the environment than a frame of data captured by a range sensor. Only a fraction of the scenes captured in a satellite map is present in a ground sensor field of view, resulting in the scan to appear drastically different depending on the sensor pose. In other words, the mapping from a satellite image to a range sensor image is not one-to-one, but one-to-many, as illustrated in Figure 3. Figure 4 demonstrates this concept on real data: the overlapping regions of the two satellite images are identical, whereas the two radar images observe different portions of the scene and as such appear drastically different.

**Fig. 5.**
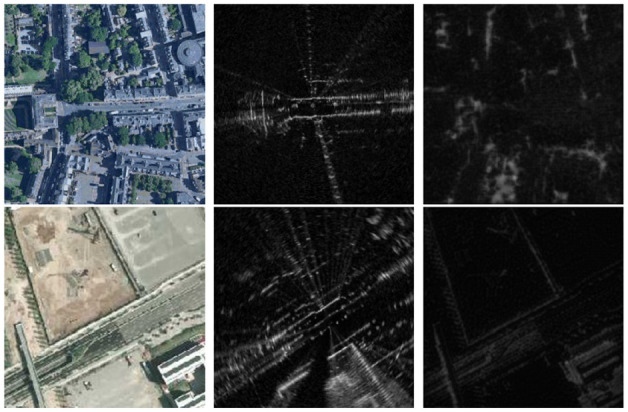
Results of CycleGAN: satellite image (left), live radar image pixel-wise aligned with the satellite image (middle), synthetic radar image (right). There is no explicit constraint on which regions of the input satellite image will appear in the output synthetic image. As a result, this leads to large localization error as the synthetic image does not contain scenes observed by the live radar image.

By using a naive image-to-image transfer approach, there is no guarantee for the generated image to contain regions of the scene that are useful for pose comparison against the live data image. Figure 5 shows examples of images generated using CycleGAN (Zhu et al., 2017), where the synthetic image highlights different scenes than what are observed by the live data image. The issue with observability or occlusion can potentially be handled by ray-tracing, such as in Kaminsky et al. (2009). However, not only is this computationally expensive, it does not apply to FMCW radars which have multiple range returns per azimuth (see Barnes et al. (2020) and Cen and Newman (2019) for more details on the sensing characteristics of FMCW radars).

Our approach inherently addresses this problem: by conditioning the image generation with the live data image, we can encourage the synthetic image to capture regions of the scene also observed by the live data image, as shown in Sections 4 and 6. This concept is analogous to learning the mapping on the right of Figure 3, where, by using a pair of satellite and range sensor images as input, the regions of the scene to be present in the output synthetic image is no longer ambiguous, but constrained by the input range sensor image.

## 4. Self-supervised cross-modality localization

Our localization pipeline is composed of three steps: rotation inference, image generation, and pose estimation. We discuss them in detail in this section.

### 4.1. Rotation inference

Given a paired map (e.g., satellite) image A∈A and live data (e.g., radar or lidar) image B∈B pre-scaled to have the same resolution but with an unknown SE(2) offset, we seek to generate a synthetic image that contains the same appearance and observed scenes as B, but is pixel-wise aligned with A.

Let the SE(2) pose difference between A and B be parametrized as ${\left[\begin{array}{ccc} x & y & \theta \end{array}\right]}^{T},$ such that by rotating B by θ and then translating by ${\left[\begin{array}{cc} x & y \end{array}\right]}^{T},$ one can pixel-wise align B onto A. The image generation can be formulated as

(1) $$f(A,B)\to {\tilde{B}}_{\theta ,\alpha }$$

where $\alpha ={\left[\begin{array}{cc} x & y \end{array}\right]}^{T}.$ Here ${\tilde{B}}_{\theta ,\alpha }$ is a generated image of modality B that synthesizes the input live sensor image B applied with a rotation of θ, followed by a translation of $\alpha ={\left[\begin{array}{cc} x & y \end{array}\right]}^{T}.$ Thus, ${\tilde{B}}_{\theta ,\alpha }$ is pixel-wise aligned with the input map image A, but contains the same observed scenes as B.

However, as originally noted by Tang et al. (2020b), the mapping in (1) is difficult to learn as the inputs A and B are offset by both a translation and a rotation. CNNs are inherently *equivariant*^1^ to translation, but not to rotation (Lenc and Vedaldi, 2015). As a result, the CNNs in the network cannot automatically utilize their mutual information and thereby capture their geometric relationship.

The method in Tang et al. (2020b) proposes to infer the rotation prior to image generation, namely, reducing (1) to two steps:

(2) $$f_{R}(A,B)\to B_{\theta }$$

(3) $$f_{G}(A,B_{\theta })\to {\tilde{B}}_{\theta ,\alpha }$$

Here $f_{R}$ is a function that infers the rotation offset θ between A and B, and outputs $B_{\theta },$ which is input image B rotated by θ. Now, $B_{\theta }$ is rotation-aligned with the map frame, and therefore offset with A only by a translation, which CNNs can naturally handle. $f_{G}$ is an image generation function that produces the synthetic image ${\tilde{B}}_{\theta ,\alpha }.$ The experiments in Tang et al. (2020b) show that learning (2) and (3) sequentially resulted in better performance than learning (1) directly, as the former is congruous with the equivariance properties of CNNs.

**Fig. 6.**
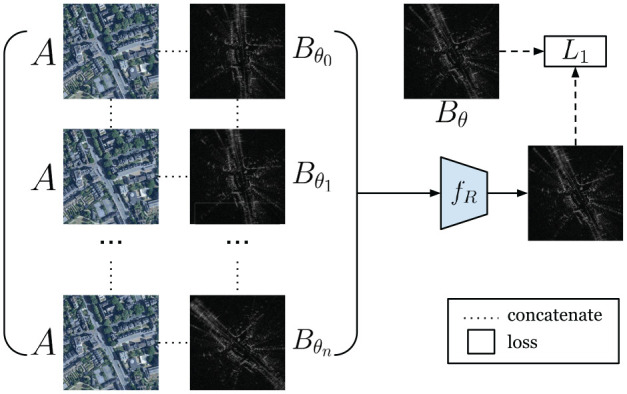
Prior work in Tang et al. (2020b) proposes a network to infer the rotation offset. The rotation offset is found by softmaxing a stack of rotated radar images to produce a radar image with the same heading as the satellite image.

In Tang et al. (2020b), the rotation inference function $f_{R}$ is parametrized by a deep network as shown in Figure 6, where satellite imagery and radar images are used as an example. Given a coarse initial heading estimate, the live data image B is rotated a number of times with small increments to form a stack of rotated images $\left\{B\}=\{B_{\theta_{0}},B_{\theta_{1}},\ldots ,B_{\theta_{n}}\right\}$ , where the number of rotations n and the increment are design parameters. Each rotated image is further concatenated with the map image to form a stacked tensor input of n pairs of map and live data images. The network assigns a latent score per input pair, and outputs a softmaxed image from {B} where the softmax weights are a function of learned latent scores:

(4) $$f_{R}(A,B_{\theta_{0}},B_{\theta_{1}},\ldots ,B_{\theta_{n}})=\sum_{i}\frac{e^{z_{i}}}{\sum_{i}e^{z_{i}}}B_{\theta_{i}}$$

where each $z_{i}$ is the associated learned scalar latent score for the pair $\{A,B_{\theta_{i}}\}.$

A loss function enforces the output to correspond to B rotated to be rotation-aligned with A, namely $B_{\theta }.$ The core idea is that the network $f_{R}$ will assign a large softmax weight to the image from {B} whose heading most closely aligns with the map image A, and small weights to all other images in {B} .

If a metrically accurate heading ground truth θ is available, then one can rotate B to form a ground-truth image target to $B_{\theta }$ used for supervising the rotation inference, as in Figure 6. In this work, however, we assume this is never the case, thus the network for $f_{R}$ must learn to infer the rotation offset in a self-supervised fashion.

For this reason, while following the same architecture as Tang et al. (2020b), our method for inferring rotation uses a different training strategy that enables self-supervised learning. In order for the network $f_{R}$ to produce the correct output, it must be able to infer the rotation from the solution space {B}, despite the modality difference between map image A and live data image B. We make the observation that if the network can infer the rotation offset from a stack of rotated live data images {B} , then, given a live data image $B_{\theta_{i}}$ , $f_{R}$ should also be able to output $A_{\theta_{i}}$ from a stack of rotated map images {A}, where $A_{\theta_{i}}$ is rotation-aligned with $B_{\theta_{i}}.$ Specifically, if we have $B_{\theta_{i}}=B_{\theta },$ then the softmaxed map image from {A} should be A, as A and $B_{\theta }$ are rotation-aligned.

As such, to learn rotation inference self-supervised, we need to pass through the network $f_{R}$ twice. The first pass is identical as in the supervised approach in Figure 6, where we denote the output softmaxed image as $B_{\theta_{i}}.$ Then $B_{\theta_{i}}$ is used as input to the second pass through network $f_{R},$ together with a stack of map images $\{A\}=\{A,A_{\phi_{0}},A_{\phi_{1}},\ldots ,A_{\phi_{m}}\}.$ The rotation angles $\left[\begin{array}{cccc} \phi_{0} & \phi_{1} & \ldots & \phi_{m} \end{array}\right]$ can be chosen randomly, and the order of {A} is shuffled such that the original non-rotated map image A can be at any index within {A}. Each image is concatenated with $B_{\theta_{i}}$ to form the input stack for passing through $f_{R}$ the second time. Note that the same network $f_{R}$ is used in both passes.

**Fig. 7.**
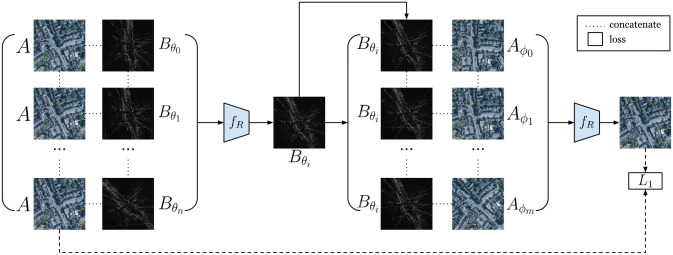
Given A and a rotation stack {B} the network $f_{R}$ finds $B_{\theta_{i}}$ by taking softmax. Then, given $B_{\theta_{i}}$ and a rotation stack {A}, the network outputs a softmaxed map image from {A}. A loss is applied to enforce the output of the second pass to be A, which in turn enforces the output of the first pass to be $B_{\theta }.$ Here both symbols for $f_{R}$ in the figure refer to the same network with the same parameters, but at different forward passes.

The network is supervised with an $L_{1}$ loss that enforces the output of the second pass to be the non-rotated map image A :

(5) $$B_{\theta_{i}}=f_{R}(A,\{B\})$$

(6) $$L_{L1}(f_{R})=E_{A,\{B\},B_{\theta_{i}}}[\|A-f_{R}(B_{\theta_{i}},\{A\}){\|}_{1}]$$

where $B_{\theta_{i}}$ is the output of the first pass. Minimizing the loss in (6) in turn enforces $B_{\theta_{i}}$ to be $B_{\theta },$ as $B_{\theta }$ is rotation-aligned with A. Our approach is shown in Figure 7. We use an increment of $2^{{}^{\circ}}$ when forming the rotation stack {B}. We evaluate the effect of the rotation increment on solution error in Section 6.10 and justify our choice.

The estimate for the rotation offset, $\hat{\theta },$ can then be found from the arg-softmax for the rotation stack {B}.

### 4.2. Image Generation

**Fig. 8.**
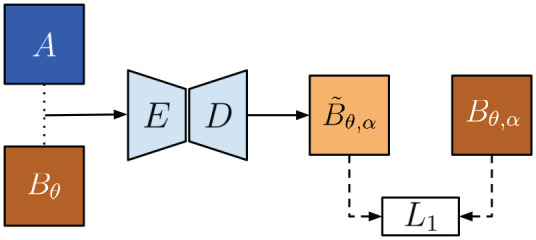
Architecture for image generation in prior supervised approach (Tang et al., 2020b).

Given A and $B_{\theta }$ we seek to generate a synthetic image ${\tilde{B}}_{\theta ,\alpha }$ as in (3), where ${\tilde{B}}_{\theta ,\alpha }$ is pixel-wise aligned with A. Tang et al. (2020b) learns the image generation function by a supervised approach, concatenating A and $B_{\theta },$ and applying an encoder–decoder architecture, as shown in Figure 8. This is possible because a target for the synthetic image ${\tilde{B}}_{\theta ,\alpha }$ can be obtained by applying the ground-truth transform.

In the supervised approach in Tang et al. (2020b), a loss can be formed between the synthetic image and the target:

(7) $$L_{L1}(f_{G})=E_{A,B_{\theta },B_{\theta ,\alpha }}[\|B_{\theta ,\alpha }-f_{G}(A,B_{\theta }){\|}_{1}]$$

where $B_{\theta ,\alpha }$ is the target with ground truth transform.

**Fig. 9.**
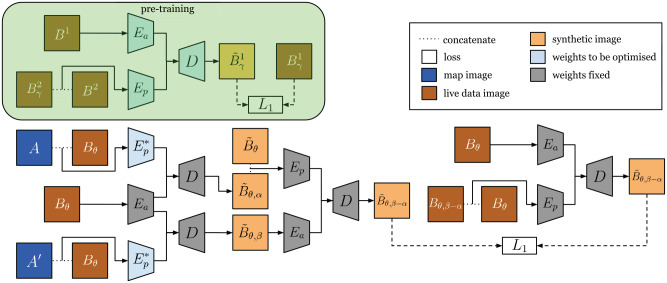
Top: During pre-training, we can learn an appearance encoder $E_{a},$ and a pose encoder $E_{p}$ that discovers the translation offset between an image of B and a shifted version of itself. Bottom: Taking $E_{a},$ $E_{p},$ and D and fixing their weights, we seek to learn $E_{p}^{*}$ which discovers the translation offset between two images from different modalities. Here $E_{a},$ $E_{p},$ and D can provide the necessary geometric and appearance relationships used for learning $E_{p}^{*}$ self-supervised.

To generate synthetic images self-supervised, we propose an architecture we call PASED, shown in Figure 9. PASED is trained in two steps: the first is a pre-training, intra-modality process that can be supervised with ground truth image targets (top half of Figure 9), whereas the second handles cross-modality comparison (bottom half of Figure 9).

#### 4.2.1. Pre-training step

Taking two random images $B^{1}$ and $B^{2}$ in the live data modality B from the training set, where $B^{1}$ and $B^{2}$ can be at arbitrary heading, we apply a known translation offset $\gamma \in R^{2}$ to $B^{2}.$ This forms an image $B_{\gamma }^{2}$ that is a shifted version of $B^{2}.$ We pass $B^{1}$ through an appearance encoder $E_{a}$ that encodes its appearance and observed scenes. Then $B_{\gamma }^{2}$ and $B^{2}$ are passed as inputs to a pose encoder $E_{p}$ that encodes the translation offset between the input images. The latent spaces from $E_{a}$ and $E_{p}$ are combined before passing through a decoder D, which outputs a synthetic image ${\tilde{B}}_{\gamma }^{1}$ that is $B^{1}$ shifted by a translation γ :

(8) $${\tilde{B}}_{\gamma }^{1}=D(E_{a}(B^{1}),E_{p}(B_{\gamma }^{2},B^{2}))$$

In other words, PASED discovers the translation offset between the two images passed as input to $E_{p},$ and applies the latent translation encoding to the input image of $E_{a}.$ The pre-training can be supervised as γ is known, thus we can shift $B^{1}$ by γ to produce the target $B_{\gamma }^{1}.$ The loss function can be formulated as

(9) $$L_{L1}(E_{a},E_{a},D)=E_{B^{1},B^{2},B_{\gamma }^{1},B_{\gamma }^{2}}\left[\|B_{\gamma }^{1}-{\tilde{B}}_{\gamma }^{1}{\|}_{1}\right]$$

and the parameters for networks $E_{a},E_{p},$ and D can be optimized by minimizing (9).

The fact that we use different images $B^{1}$ and $B^{2}$ for inputs to $E_{a}$ and $E_{p}$ ensures appearance and pose are disentangled from each other. As shown later, this allows modules of PASED to be separated and re-combined with newly learned modules.

#### 4.2.2. Cross-modality step

In the second step, we fix the weights of $E_{a},$ $E_{p},$ and D which are optimized from the pre-training step. This narrows down the self-supervision problem to learning a cross-modality pose encoder $E_{p}^{*}$ that discovers the translation offset between an image of modality A and another of B. Taking A and $B_{\theta }$ as inputs, $E_{p}^{*}$ should encode the unknown translation offset α between them. Concurrently, $B_{\theta }$ is fed to $E_{a}$ to encode its appearance and the resulting latent space is combined with the latent space produced by $E_{p}^{*}(A,B_{\theta }),$ before being decoded by D. This encoder–decoder combination will generate a synthetic image ${\tilde{B}}_{\theta ,\alpha },$ which we do not have a target for:

(10) $${\tilde{B}}_{\theta ,\alpha }=D(E_{a}(B_{\theta }),E_{p}^{*}(A,B_{\theta }))$$

We can apply a known shift to the center position of A to query another map image A′, where A′ is offset with $B_{\theta }$ by an unknown translation β. Using the same encoder–decoder combination as before, we can take A′ and $B_{\theta }$ to generate a synthetic image ${\tilde{B}}_{\theta ,\beta }:$

(11) $${\tilde{B}}_{\theta ,\beta }=D(E_{a}(B_{\theta }),E_{p}^{*}(A\prime ,B_{\theta }))$$

Furthermore, given $B_{\theta }$ and the networks learned from pre-training, we can easily generate ${\tilde{B}}_{\theta }$ by encoding a zero shift:

(12) $${\tilde{B}}_{\theta }=D(E_{a}(B_{\theta }),E_{p}(B_{\theta },B_{\theta }))$$

If we pass ${\tilde{B}}_{\theta }$ and ${\tilde{B}}_{\theta ,\alpha }$ to the pre-trained pose encoder $E_{p},$ then the latent space will encode a shift of −α. Combing this latent space with $E_{a}({\tilde{B}}_{\theta ,\beta }),$ we can decode a synthetic image ${\tilde{B}}_{\theta ,\beta -\alpha }:$

(13) $${\tilde{B}}_{\theta ,\beta -\alpha }=D(E_{a}({\tilde{B}}_{\theta ,\beta }),E_{p}({\tilde{B}}_{\theta },{\tilde{B}}_{\theta ,\alpha }))$$

Here β−α is a known value as it is the translation offset applied to A to obtain A′.

We can shift $B_{\theta }$ by β−α to obtain $B_{\theta ,\beta -\alpha }.$ Using $B_{\theta ,\beta -\alpha }$ and $B_{\theta },$ we can generate ${\tilde{B}}_{\theta ,\beta -\alpha }$ with pre-trained networks $E_{a},$ $E_{p},$ and D, shown on the bottom right of Figure 9. Specifically, this can be expressed as

(14) $${\tilde{B}}_{\theta ,\beta -\alpha }=D(E_{a}(B_{\theta }),E_{p}(B_{\theta ,\beta -\alpha },B_{\theta }))$$

We can form ${\tilde{B}}_{\theta ,\beta -\alpha }$ using two different combinations of inputs as in (13) and (14). Notably, ${\tilde{B}}_{\theta ,\beta -\alpha }$ formed using (14) only passes through networks with weights optimized from the pre-training step and fixed during the cross-modality step, and therefore can be used as a target. A loss can then be established between the two synthetic images from (13) and (14), where the latter is a target image:

(15) $$\begin{array}{c} L_{L1}(E_{p}^{*})=E_{A,A\prime ,B_{\theta },B_{\theta ,\beta -\alpha }}[∥D(E_{a}(B_{\theta }),E_{p}(B_{\theta ,\beta -\alpha },B_{\theta }))\\ -D(E_{a}({\tilde{B}}_{\theta ,\beta }),E_{p}({\tilde{B}}_{\theta },{\tilde{B}}_{\theta ,\alpha })){∥}_{1}] \end{array}$$

By back-propagation, the loss in (15) optimizes the network $E_{p}^{*}$ , as ${\tilde{B}}_{\theta ,\alpha }$ and ${\tilde{B}}_{\theta ,\beta }$ are functions of $E_{p}^{*}.$ Alternatively we can use $B_{\theta ,\beta -\alpha }$ as the target, but, in practice, using ${\tilde{B}}_{\theta ,\beta -\alpha }$ as in (14) led to faster convergence.

For the loss in (15) to be minimized, two conditions must hold true. First, ${\tilde{B}}_{\theta ,\beta }$ must have correctly encoded the appearance and observed scenes in $B_{\theta }.$ Second, ${\tilde{B}}_{\theta ,\alpha }$ and ${\tilde{B}}_{\theta ,\beta }$ must have the correct translations α and β, respectively. By satisfying these two constraints we can ensure $E_{p}^{*}$ is able to discover the translation offset across modalities, and is compatible with pre-trained networks $E_{a}$ and D for image generation.

### 4.3. Pose estimation

Taking the pose-aligned synthetic image ${\tilde{B}}_{\theta ,\alpha }$ and the rotation-aligned range sensor image $B_{\theta },$ we embed them to a joint space, where their translation offset is found by maximizing correlation on the learned embeddings. We denote the embedding network for real and synthetic images to be $H_{B}$ and $H_{\tilde{B}},$ respectively, and the learned embeddings to be $B_{\theta }^{†}$ and ${\tilde{B}}_{\theta ,\alpha }^{†}:$

(16) $$B_{\theta }^{†}=H_{B}(B_{\theta }),\;{\tilde{B}}_{\theta ,\alpha }^{†}=H_{\tilde{B}}({\tilde{B}}_{\theta ,\alpha })$$

The correlation maximization is a parameter-free process that requires no additional learned modules, but is differentiable allowing gradients induced by the downstream loss to propagate to upstream learned modules. In this step, we can infer $\hat{\alpha }={\left[\begin{array}{cc} \hat{x} & \hat{y} \end{array}\right]}^{T},$ which is our posterior estimate to the translation. Formally, this can be expressed as

(17) $$\hat{\alpha }=\underset{p\in {\mathbb{R}}^{2}}{\mathrm{argmax}}{\tilde{B}}_{\theta ,\alpha }^{†}★B_{\theta }^{†}$$

where ${\tilde{B}}_{\theta ,\alpha }^{†}★B_{\theta }^{†}$ is the discrete cross-correlation between ${\tilde{B}}_{\theta ,\alpha }^{†}$ and $B_{\theta }^{†}.$ This can be performed efficiently in the Fourier domain, as is done in prior works that use a similar approach (Barnes et al., 2019; Barsan et al., 2018; Tang et al., 2020b).

**Fig. 10.**
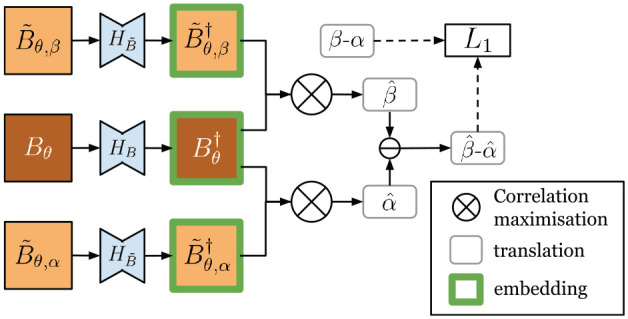
The networks $H_{B}$ and $H_{\tilde{B}}$ are learned to project real live images and synthetic images to a joint embedding, where their translation offset can be found by maximizing correlation.

The embeddings are thus learned to further ensure the synthetic image and the live image can be correlated correctly. Without ground truth α, we can self-supervise using a similar approach as in learning PASED, by applying a known shift. The architecture for learning the embeddings is shown in Figure 10. Similar as in Section 4.2, ${\tilde{B}}_{\theta ,\beta }$ can be obtained by shifting the map image A to obtain A′. Given learned deep embeddings ${\tilde{B}}_{\theta ,\beta }^{†}$ and $B_{\theta }^{†},$ the translation offset by correlation maximization is found to be $\hat{\beta }:$

(18) $${\tilde{B}}_{\theta ,\beta }^{†}=H_{\tilde{B}}({\tilde{B}}_{\theta ,\beta })$$

(19) $$\hat{\beta }=\underset{p\in R^{2}}{\arg\;\max}{\tilde{B}}_{\theta ,\beta }^{†}★B_{\theta }^{†}$$

The difference of the two offsets β−α is known, and can be used to establish a loss term:

(20) $$L_{L1}(H_{B},H_{\tilde{B}})=E_{B_{\theta },{\tilde{B}}_{\theta ,\alpha },{\tilde{B}}_{\theta ,\beta }}[\|\beta -\alpha -(\hat{\beta }-\hat{\alpha }){\|}_{1}]$$

**Fig. 11.**
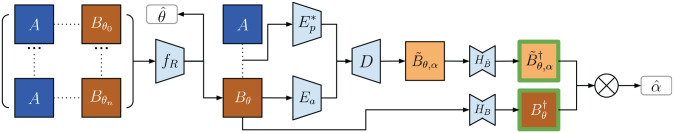
Overall data flow of our method at inference: given map image A and live data image B, based on the initial heading estimate, we form a stack of rotated images $\{B_{\theta_{0}},\ldots ,B_{\theta_{n}}\},$ from which $f_{R}$ discovers $B_{\theta }$ that is B rotated to be rotation-aligned with A. This process also infers the heading estimate $\hat{\theta }.$ Here A and $B_{\theta }$ are used to generate a synthetic image ${\tilde{B}}_{\theta ,\alpha }$ that has the same appearance and observed scene as $B_{\theta }$ and is pose-aligned with A ; and ${\tilde{B}}_{\theta ,\alpha }$ and $B_{\theta }$ are projected to deep embeddings ${\tilde{B}}_{\theta ,\alpha }^{†}$ and $B_{\theta }^{†},$ where the estimate for the translation offset $\hat{\alpha }$ is found by correlation maximization.

The overall pipeline for data flow at inference time is shown in Figure 11.

## 5. Implementation details

Here we provide details on the architecture of the various networks used in our method, and the associated hyper-parameters. We make use of the following abbreviations.

- RP( p ): 2D reflection padding of p .
- Conv( $C_{\mathrm{in}},C_{\mathrm{out}},k,s,p$ ): convolution with $C_{\mathrm{in}}$ input channels, $C_{\mathrm{out}}$ output channels, kernel size k, stride s, padding p, and bias.
- IN: instance normalization.
- ReLU: rectified linear unit.
- LReLU( m ): leaky ReLU with negative slope m .
- Drop( d ): dropout with ratio d .
- ConvT( $C_{\mathrm{in}},C_{\mathrm{out}},k,s,p,p_{\mathrm{out}}$ ): transposed convolution with $C_{\mathrm{in}}$ input channels, $C_{\mathrm{out}}$ output channels, kernel size k, stride s, padding p, output padding $p_{\mathrm{out}},$ and bias.

The network architectures are listed in Tables 1 to 4. For comparison against the prior supervised approach, we use the same architectures where possible. We implemented the image generation network for the prior supervised approach to have the same latent space size at the bottleneck, and the same number of down-sample and up-sample layers as in our method.

**Table 1.** Architecture for rotation inference.

Rotation Inference Function $f_{R}$
Input shape: n×4×256×256 where C=4, H=W=256
Conv(4, 32, 3, 2, 1) + IN + ReLU
Conv(32, 64, 3, 2, 1) + IN + ReLU
Conv(64, 128, 3, 2, 1) + IN + ReLU
Conv(128, 256, 3, 2, 1) + IN + ReLU
Latent shape: n×256×16×16
Take the mean along C,H,W + Softmax + Reshape
Latent vector shape: 1×n, which are the softmax weights
Matrix-multiple softmax weights with the input
Shape of the multiplication product: 4×256×256
Extract the associated channel(s) to get $B_{\theta }$ (or $A_{\theta_{i}}$ during training)

**Table 2.** Architecture of for image generation.

Appearance Encoder $E_{a}$
RP(3) + Conv(1, 16, 7, 1, 0) + IN + ReLU
Conv(16, 32, 3, 2, 1) + IN + ReLU
Conv(32, 64, 3, 2, 1) + IN + ReLU
Conv(64, 128, 3, 2, 1) + IN + ReLU
Conv(128, 256, 3, 2, 1) + IN + ReLU
ResNet blocks ( ×9 ):
Conv(256, 256, 3, 1, 0) + IN + ReLU + Drop(0.5)
Conv(256, 256, 3, 1, 0) + IN
Intra-Modality Pose Encoder $E_{p}$
RP(3) + Conv(2, 16, 7, 1, 0) + IN + ReLU
Conv(16, 32, 3, 2, 1) + IN + ReLU
Conv(32, 64, 3, 2, 1) + IN + ReLU
Conv(64, 128, 3, 2, 1) + IN + ReLU
Conv(128, 256, 3, 2, 1) + IN + ReLU
ResNet blocks ( ×9 ):
Conv(256, 256, 3, 1, 0) + IN + ReLU + Drop(0.5)
Conv(256, 256, 3, 1, 0) + IN
Cross-Modality Pose Encoder $E_{p}^{*}$
RP(3) + Conv(4, 16, 7, 1, 0) + IN + ReLU
Conv(16, 32, 3, 2, 1) + IN + ReLU
Conv(32, 64, 3, 2, 1) + IN + ReLU
Conv(64, 128, 3, 2, 1) + IN + ReLU
Conv(128, 256, 3, 2, 1) + IN + ReLU
ResNet blocks ( ×9 ):
Conv(256, 256, 3, 1, 0) + IN + ReLU + Drop(0.5)
Conv(256, 256, 3, 1, 0) + IN
Decoder D
ConvT(512, 256, 3, 2, 1, 1) + IN + ReLU + Drop(0.5)
ConvT(256, 128, 3, 2, 1, 1) + IN + ReLU + Drop(0.5)
ConvT(128, 64, 3, 2, 1, 1) + IN + ReLU + Drop(0.5)
ConvT(64, 32, 3, 2, 1, 1) + IN + ReLU + Drop(0.5)
RP(3) + Conv(32, 1, 7, 1, 0) + Sigmoid

**Table 3.** Image generation for our implementation of RSL-Net for comparison.

Encoder E
RP(3) + Conv(4, 32, 7, 1, 0) + IN + ReLU
Conv(32, 64, 3, 2, 1) + IN + ReLU
Conv(64, 128, 3, 2, 1) + IN + ReLU
Conv(128, 256, 3, 2, 1) + IN + ReLU
Conv(256, 512, 3, 2, 1) + IN + ReLU
ResNet blocks ( ×9 ):
Conv(512, 512, 3, 1, 0) + IN + ReLU + Drop(0.5)
Conv(512, 512, 3, 1, 0) + IN
Decoder D
ConvT(512, 256, 3, 2, 1, 1) + IN + ReLU + Drop(0.5)
ConvT(256, 128, 3, 2, 1, 1) + IN + ReLU + Drop(0.5)
ConvT(128, 64, 3, 2, 1, 1) + IN + ReLU + Drop(0.5)
ConvT(64, 32, 3, 2, 1, 1) + IN + ReLU + Drop(0.5)
RP(3) + Conv(32, 1, 7, 1, 0) + Sigmoid

**Table 4.** U-Net architecture for learning embeddings.

Embedding Networks $H_{B}$ and $H_{\tilde{B}}$
Conv(1, 32, 4, 2, 0)
LReLU(0.2) + Conv(32, 64, 4, 2, 0) + IN
LReLU(0.2) + Conv(64, 128, 4, 2, 0) + IN
LReLU(0.2) + Conv(128, 256, 4, 2, 0) + IN
LReLU(0.2) + Conv(256, 512, 4, 2, 0) + IN
LReLU(0.2) + ReLU + Conv(512, 1024, 4, 2, 0)
ReLU + ConvT(1024, 512, 4, 2, 1, 0) + IN
ReLU + ConvT(512, 256, 4, 2, 1, 0) + IN
ReLU + ConvT(256, 128, 4, 2, 1, 0) + IN
ReLU + ConvT(128, 64, 4, 2, 1, 0) + IN
ReLU + ConvT(64, 32, 4, 2, 1, 0) + IN
ReLU + ConvT(32, 1, 4, 2, 1, 0) + Sigmoid
With skip connections in-between intermediate layers

Our method is implemented in PyTorch (Paszke et al., 2019). For training rotation inference $f_{R}$ and networks for image generation $E_{a},$ $E_{p},$ $E_{p}^{*},$ and D, we use a learning rate of $2e^{-4}.$ For learning the embedding networks $H_{B}$ and $H_{\tilde{B}},$ we use a learning rate of $2\times 10^{-6}.$ We use Adam (Kingma and Ba, 2015) as the optimizer for all experiments. The training is terminated when the validation loss increases for more than five epochs, or reaching a maximum number of epochs. This results in 80 to 150 epochs of training for learning $f_{R},$ $E_{a},$ $E_{p},$ $E_{p}^{*},$ and D, depending on the dataset and the specific experiment, and 10 to 20 epochs for learning $H_{B}$ and $H_{\tilde{B}}.$ The inference runs at about 10 Hz. On a single 1080 Ti GPU. We use a batch size of 32 for all experiments unless otherwise stated.

## 6. Experimental validation

We evaluate on several public, real-world datasets collected with vehicles equipped with on-board range sensors. The datasets we use come with metric ground truths that are decently accurate, though we noticed the GPS/INS solutions in certain places can drift up to a few meters.

We add large artificial pose offsets to the ground truth when querying for a satellite image, thereby simulating a realistic robot navigation scenario where the initial pose estimate can solve place recognition, but is too coarse for the robot’s metric pose. Using a map (e.g., satellite) image queried at this coarse initial pose estimate, our method solves metric localization by comparing against the live sensor data. The true pose offsets are hidden during training as our method is self-supervised, and are only revealed at test time for evaluation purposes.

The artificial offset is chosen such that the initial estimate has an unknown heading error in the range $[-\frac{\pi }{8},\frac{\pi }{8}],$ therefore given the initial estimate $\theta_{0},$ the rotation inference must choose a solution space of at least $\left[\theta_{0}-\frac{\pi }{8},\theta_{0}+\frac{\pi }{8}\right]$ to guarantee the correct solution can be found. We use a pixel-wise translation error in the range [−25,25] pixels. Depending on the resolution for a specific experiment, this corresponds to an error of at least [−10m,10m] and up to more than [−20m,20m]. All experiments use input images of size 256×256.

### 6.1. Radar localization against satellite imagery

We evaluate our method on two datasets with FMCW radar and GPS: the Oxford Radar RobotCar Dataset (Barnes et al., 2020) and the MulRan Dataset (Kim et al., 2020). The satellite images for RobotCar are queried using Google Maps Platform.^2^ For MulRan they are queried using Bing Maps Platform,^3^ as high-definition Google satellite imagery is unavailable at the place of interest.

The GPS/INS and range sensor data for all datasets used are timestamped. To create the ground truth, for each frame of range sensor data, we find its associated latitude, longitude, and heading from the GPS/INS data based on the time-stamp, and query a satellite image with the corresponding latitude and longitude. We also rotate the range sensor image with the ground-truth heading to generate a rotation-aligned range sensor image. To add the initial offset for simulating a coarse initial estimate, we simply shift the center of the satellite images by the translation offset and rotate the range sensor image by the heading offset when forming the range sensor–satellite pairs.

**Fig. 12.**
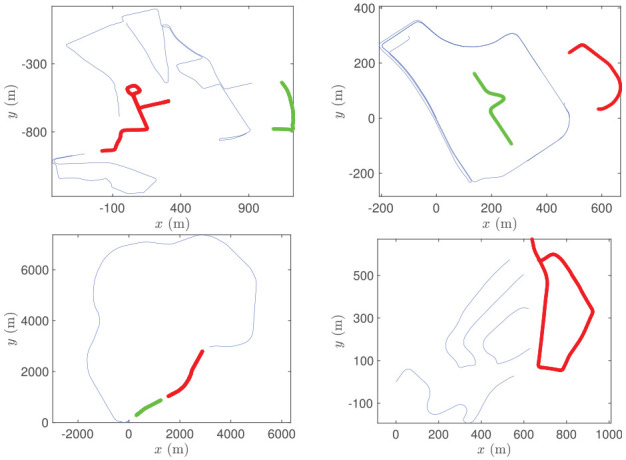
Training (blue), validation (green), and test (red) trajectories for RobotCar (top left), KAIST (top right), Sejong (bottom left) and 20111003_drive0034 (bottom right). Certain data are removed to avoid overlap between the splits.

We benchmark against the prior supervised method RSL-Net (Tang et al., 2020b) in our experiments, which is evaluated only on the RobotCar Dataset. Both datasets contain repeated traversals of the same routes. We separately train, validate, and test for every dataset, splitting the data as in Figure 12. For the RobotCar Dataset, we split the trajectories the same way as in Tang et al. (2020b) for a fair comparison. For the RobotCar Dataset, the training set consists of training data from sequences no. 2, no. 5, and no. 6, whereas we test on the test data from sequence no. 2. For the MulRan Dataset, we used sequences KAIST 01 and Sejong 01. The resulting test sets feature an urban environment (RobotCar), a campus (KAIST 01) and a highway (Sejong 01).

We test on every fifth frame, resulting in 201 frames from the RobotCar Dataset and 358 from the MulRan Dataset, spanning a total distance of almost 4 km. The resolution used is 0.8665m/pixel for RobotCar and 0.7876m/pixel for MulRan. All sensor data are pre-scaled to have the same resolution as satellite images. The mean errors and standard deviations around the mean are reported in Table 5. Starting from a large initial offset, we can localize to an error of a few meters in translation and a few degrees in heading. Our method achieves an error on par with the supervised approach in Tang et al. (2020b), while requiring no metrically accurate ground truth for training.

**Table 5.** Mean error and error standard deviation for radar localization against satellite imagery.

	**Mean error (metric)**			**Mean error (pixel)**		**Error STD (metric)**		
	x(m)	y(m)	θ(°)	x	y	x(m)	y(m)	θ(°)
RobotCar (ours)	3.44	5.40	**3.03**	3.97	6.23	4.44	7.50	**3.04**
RobotCar (RSL-Net (Tang et al., 2020b), supervised)	**2.74**	**4.26**	3.12	**3.16**	**4.92**	**4.32**	**6.48**	4.16
MulRan (ours)	6.02	**7.02**	2.92	7.64	**8.91**	**5.75**	6.87	3.64
MulRan (RSL-Net)	**5.85**	7.11	**1.88**	**7.42**	9.03	6.50	**6.46**	**2.41**

### 6.2. Lidar localization against satellite imagery

For this experiment, we evaluate on the RobotCar Dataset (Barnes et al., 2020) which also has two Velodyne HDL-32E lidars mounted in a tilted configuration, and KITTI (raw dataset (Geiger et al., 2013)) which has a Velodyne HDL-64E lidar and GPS data.

For the RobotCar Dataset, the trajectories are split into training, validation, and test sets approximately the same way as in Section 6.1. For the KITTI Dataset, the training set includes sequences 20110929_drive0071, 20110930_drive0028, and 20111003_drive0027. Sequence 20110926_drive0117 is used for validation. Finally, data in 20111003_drive0034 are split into training and test, as shown in Figure 12. To turn 3D lidar point-clouds to lidar images, the point-clouds are projected to the x – y plane. We discard points with z values smaller than zero to remove ground points when creating the lidar images. Although such a simple approach may result in certain non-ground points removed, we observe that such an effect is rather minimal. The resulting lidar images are grey-scale images where pixel values are the normalized intensity.

As lidars have a shorter range than radars, we use satellite images of a greater zoom level, with resolution 0.4332m/pixel for RobotCar and 0.4592m/pixel for KITTI. The test set consists of 200 frames for RobotCar and 253 for KITTI, spanning a total distance of near 3km. The test set for KITTI features a residential area. The results are reported in Table 6. The error on the RobotCar Dataset is smaller when using lidar for localization than when using radar.

**Table 6.** Mean error and error standard deviation for lidar localization against satellite imagery.

	**Mean error (metric)**			**Mean error (pixel)**		**Error STD (metric)**		
	x(m)	y(m)	θ(°)	x	y	x(m)	y(m)	θ(°)
RobotCar (ours)	**1.54**	**1.85**	2.29	**3.55**	**4.27**	**1.54**	**2.29**	3.10
RobotCar (RSL-Net)	2.31	2.55	**2.08**	5.33	5.89	2.26	2.57	**2.91**
KITTI (ours)	3.05	3.13	1.67	6.64	6.82	**3.59**	**3.78**	2.57
KITTI (RSL-Net)	**2.45**	**2.79**	**1.59**	**5.34**	**6.08**	3.70	3.88	**2.22**

### 6.3. Radar localization against prior lidar map

Though our method is designed for localizing against satellite imagery, we show it can also handle more standard forms of cross-modality localization. Here we build a lidar map using a prior traversal, and localize using radar from a later traversal.

We demonstrate on the RobotCar and MulRan datasets, where we use the same resolution as in Section 6.1. For RobotCar, we use ground truth to build a lidar map from sequence no. 2. Radar data in the training sections from no. 5 and no. 6 as in Figure 12 form the training set, whereas the test section from sequence no. 5 forms the test set. For MulRan, lidar maps are built from KAIST 01 and Sejong 01, and we localized using radar data from KAIST 02 and Sejong 02, which are split into training, validation, and test sets. This resulted in a test set consisting of 201 frames from RobotCar and 272 frames from MulRan, spanning a total distance of almost 4 km. The localization results are Table 7.

**Table 7.** Mean error and error standard deviation for radar localization against prior lidar map.

	**Mean error (metric)**			**Mean error (pixel)**		**Error STD (metric)**		
	x(m)	y(m)	θ(°)	x	y	x(m)	y(m)	θ(°)
RobotCar (ours)	**2.21**	**2.57**	2.65	**2.55**	**2.97**	**2.30**	3.53	**2.06**
RobotCar (RSL-Net)	2.66	3.41	**2.45**	3.07	3.93	2.85	**3.11**	2.18
RobotCar (CycleGAN)	6.41	9.05	2.65	7.40	10.44	7.43	7.17	2.06
MulRan (ours)	3.57	3.26	2.15	4.53	4.13	4.29	4.84	2.38
MulRan (RSL-Net)	**3.37**	**2.61**	**1.40**	**4.28**	**3.32**	**3.38**	**4.12**	**1.71**
MulRan (CycleGAN)	4.84	4.39	2.15	6.14	5.58	5.78	4.96	2.38

This experiment is more suitable for naive image generation methods such as CycleGAN (Zhu et al., 2017) than previous experiments, because the field of view is considerably more compatible as both modalities are from range sensors. In Table 7, we list results where we replaced the image generation stage of our method by CycleGAN while keeping other modules unchanged. The localization results are, however, much worse when modality A is satellite imagery, as shown qualitatively in Figure 5.

### 6.4. Online pose-tracking system

In prior experiments we assumed place recognition is always available, providing a coarse initial estimate for every frame. Here we present a stand-alone pose-tracking system by continuously localizing against satellite imagery. Given a coarse initial estimate (e.g., from GPS) for the first frame, the vehicle localizes and computes its pose within the satellite map. The initial estimate for every frame onward is then set to be the computed pose of the previous frame. We only need place recognition once at the very beginning; the vehicle then tracks its pose onward without relying on any other measurements.

#### 6.4.1. Introspection

As localizing using satellite imagery is challenging, the result will not always be accurate. Our method, however, naturally allows for introspection. A synthetic image ${\tilde{B}}_{\theta ,\alpha }$ was generated from A and $B_{\theta }.$ We can apply a known small translation offset δ to A to form $A_{\delta }.$ Taking $A_{\delta }$ and $B_{\theta }$ we can generate ${\tilde{B}}_{\theta ,\alpha +\delta }.$ Finally, we can compute a translation offset $\hat{\delta }$ by passing ${\tilde{B}}_{\theta ,\alpha +\delta }$ and ${\tilde{B}}_{\theta ,\alpha }$ through the learned embeddings and maximizing correlation:

(21) $$\hat{\delta }=\underset{p\in R^{2}}{\arg\;\max}{\tilde{B}}_{\theta ,\alpha +\delta }^{†}★{\tilde{B}}_{\theta ,\alpha }^{†}$$

Let $d_{\mathrm{intro}}=\|\delta -\hat{\delta }\|.$ A large value of $d_{\mathrm{intro}}$ indicates the generated images are erroneous. This allows us to examine the solution quality; our system falls back to using odometry for dead-reckoning when $d_{\mathrm{intro}}$ exceeds a threshold. We do not require high-quality odometry, but rather only use a naive approach by directly maximizing correlation between two consecutive frames without any learned modules. In our experiments, we set δ to be ${\left[\begin{array}{cc} 10 & 10 \end{array}\right]}^{T},$ and $d_{\mathrm{intro}}$ to be 5.

#### 6.4.2. Results

**Fig. 13.**
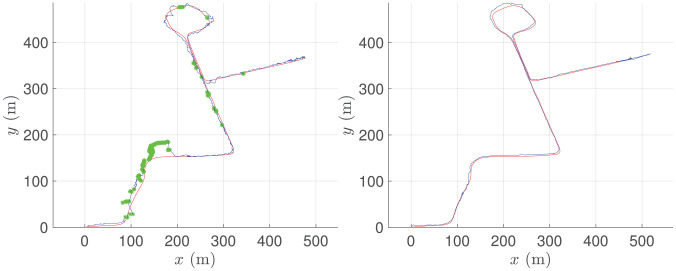
Estimated pose (blue) versus ground-truth pose (red) for localizing a radar (left) and a lidar (right) against satellite imagery. Our system tracks the vehicle’s pose over 1 km, where we occasionally fall back to odometry for the radar experiment (green). Our system is stand-alone and requires GPS only for the first frame.

We conduct two experiments on the test set of RobotCar, one where we track a radar using satellite imagery, and one where we track a lidar. For both experiments we run localization at 4 Hz. The results are shown in Figure 13. If the solution error is too large, then the initial estimate will be too off for a sufficient overlap between the next queried satellite image and live data, resulting in losing track of the vehicle. Although the solution error is large for certain frames, our system continuously localizes the vehicle for over a kilometer without completely losing track. For the lidar experiment, the solutions are sufficiently accurate to not require any odometry. Each solution only uses a single frame of data (plus the solution from the previous frame for the initial estimate), and we make no attempt at windowed/batch optimization or loop closures.

### 6.5. Ablation study

We perform an ablation study to investigate the effect of reduced training data. For radar localization against satellite imagery on the RobotCar Dataset, we trained a model using approximately the first 20% of training data, and another using every 10 ^th^ frame of training data. The results are shown in Table 8.

**Table 8.** Ablation study for using reduced training data, evaluated on radar localization against satellite imagery on the RobotCar Dataset.

	**Mean error (metric)**			**Mean error (pixel)**	
	x(m)	y(m)	θ(°)	x	y
RobotCar (full)	**3.44**	**5.40**	**3.03**	**3.97**	**6.23**
RobotCar (first 20% )	7.96	7.45	6.03	9.18	8.59
RobotCar (every 10 ^th^)	4.36	6.18	4.40	5.03	7.14

Noticeably, the choice of selecting uniformly distributed data in contrast to utilizing only the first 20% leads to a more varied dataset, and as such achieves better performances despite the lower number of samples.

### 6.6. Testing on different sequences

For the results in Tables 5, 6, and 7, the training and test data, though with zero spatial overlap, are from the same sequences. The RobotCar and MulRan datasets contain repeated sequences of the same trajectory. Here we show results where the test set trajectories are from different sequences, to demonstrate the capability of generalizing not only to unseen places, but also to range sensor data recorded on different days as the training data.

We arbitrarily selected sequences no. 7 and no. 15 as the new test sequences for RobotCar, and sequences KAIST 02 and Sejong 02 for MulRan. The same test trajectories as in Figure 12 are extracted for the new test sequences. The training trajectories and sequences remain unchanged.

The localization results are listed in Table 9 and Table 10. The mean errors are fairly consistent across different sequences.

**Table 9.** Results for radar localization against satellite imagery on multiple test sequences.

	**Mean error (metric)**			**Mean error (pixel)**		**Error STD (metric)**		
	x(m)	y(m)	θ(°)	x	y	x(m)	y(m)	θ(°)
RobotCar (sequence no. 1)	3.44	5.40	3.03	3.97	6.23	4.44	7.50	3.04
RobotCar (sequence no. 7)	3.44	5.15	3.96	3.97	5.95	5.76	5.44	4.32
RobotCar (sequence no. 15)	3.21	5.21	3.80	3.70	6.01	2.83	5.76	4.20
MulRan (KAIST01, Sejong 01)	6.02	7.02	2.92	7.64	8.91	5.75	6.87	3.64
MulRan (KAIST02, Sejong 02)	6.44	6.59	5.15	8.19	8.37	6.64	6.62	7.32

**Table 10.** Results for lidar localization against satellite imagery on multiple test sequences.

	**Mean error (metric)**			**Mean error (pixel)**		**Error STD (metric)**		
	x(m)	y(m)	θ(°)	x	y	x(m)	y(m)	θ(°)
RobotCar (sequence no. 1)	1.54	1.85	2.29	3.55	4.27	1.54	2.29	3.10
RobotCar (sequence no. 7)	1.38	1.72	2.35	3.18	3.98	1.37	1.83	2.71
RobotCar (sequence no. 15)	1.67	1.81	2.05	3.87	4.17	1.42	1.84	2.12

### 6.7. Circular initial offset

For a fair comparison against the supervised approach in Tang et al. (2020b), we assume the initial offset for both x and y is uniformly distributed and in the range [−25,25] pixels, which was also employed in Tang et al. (2020b). However, a more realistic initial offset that more closely mimics the error characteristics of a GPS would be sampling the initial offset from a circular area of radius r from the ground-truth position.

Without re-training, we evaluate the model performance where the initial translation offset is sampled uniformly from a circular area with a radius of 25 pixels, and centered at the ground truth position. The sampling for the initial heading offset remains unchanged. The localization results are summarized in Tables 11 and 12.

**Table 11.** Results for radar localization against satellite imagery using a circular translation offset range.

	**Mean error (metric)**			**Mean error (pixel)**		**Error STD (metric)**		
	x(m)	y(m)	θ(°)	x	y	x(m)	y(m)	θ(°)
RobotCar (ours)	2.83	4.49	3.57	3.39	5.39	**2.93**	5.48	3.71
RobotCar (RSL-Net)	**2.58**	**3.89**	**3.05**	**3.10**	**4.66**	3.85	**4.79**	**3.48**
MulRan (ours)	6.30	**6.98**	3.01	8.00	**8.86**	**5.22**	5.51	3.61
MulRan (RSL-Net)	**5.35**	7.58	**1.72**	**6.79**	9.62	5.25	**5.26**	**2.43**

**Table 12.** Results for lidar localization against satellite imagery using a circular translation offset range.

	**Mean error (metric)**			**Mean error (pixel)**		**Error STD (metric)**		
	x(m)	y(m)	θ(°)	x	y	x(m)	y(m)	θ(°)
RobotCar (ours)	**1.40**	**1.83**	**1.97**	**3.23**	**4.23**	**1.31**	**1.54**	**2.45**
RobotCar (RSL-Net)	1.81	2.15	2.09	4.19	4.96	1.53	1.90	3.17
KITTI (ours)	2.93	3.10	**1.30**	6.38	6.74	**3.06**	**3.01**	**1.10**
KITTI (RSL-Net)	**2.52**	**2.59**	1.50	**5.49**	**5.65**	3.49	3.09	**2.37**

### 6.8. Trade-offs on network width and depth

Here we show the effects of network width (number of channels in each layer) and depth (number of layers) on solution quality and the associated trade-offs, and justify choosing the architecture shown in Tables 1 to 4. In this experiment, we vary the width or depth of networks for rotation inference and image generation, namely $f_{R},E_{a},E_{p},E_{p}^{*},D,$ while keeping $H_{B}$ and $H_{\tilde{B}}$ unchanged. The results are listed in Table 13.

**Table 13.** Mean error for using various choices of network width and depth, evaluated on lidar localization against satellite imagery on the RobotCar Dataset.

	**Mean error (metric)**			**Mean error (pixel)**		**Number of parameters**
	x(m)	y(m)	θ(°)	x	y	(M)
RobotCar, channels $\times \frac{1}{2}$	1.57	1.93	2.58	3.62	4.45	5.23
RobotCar, ours	**1.54**	1.85	2.29	**3.55**	4.27	20.86
RobotCar, channels ×2	1.58	**1.65**	**2.27**	3.64	**3.80**	83.37
RobotCar, depth −1	1.70	2.09	2.47	3.92	4.82	5.22
RobotCar, ours	**1.54**	**1.85**	**2.29**	**3.55**	**4.27**	20.86
RobotCar, depth +1	1.56	1.98	2.39	3.60	4.57	83.41

First, we fix the network depth while halving or doubling the number of channels in each layer. When the width is reduced, the network representation power is noticeably affected, indicated by an increase in solution error in all of x,y, and θ. When the width is doubled, we achieved a slight reduction on the rotation error and overall translation error. However, the total number of network parameters increase by a factor of four when the width is doubled, greatly limiting the maximum affordable batch size, and thereby increasing the training time. As a result, we opted the architecture presented in Section 5.

Next, we keep the number of channels the same in the first layer, and study the effect of making the networks shallower or deeper (by one layer). For image generation we use an encoder–decoder architecture with stride 2 in the convolution layers (after the first layer) as shown in Table 2, thus the height and width of the representation decrease by a factor of 2 after each layer, becoming 16×16 at the bottleneck with an input of size 256×256 . Intuitively, a trade-off exists such that deeper networks theoretically have stronger representation power, but will result in reduced representation size at the bottleneck, making image reconstruction harder. The results in Table 13 suggest that our choice of network depth is optimal with noticeably smaller translation error, which is affected by the quality of image generation.

### 6.9. Choice of image resolution

In the results presented in Sections 6.1 and 6.2, we used a resolution of 0.8665m/pixel for experiments on radar localization against satellite imagery for the RobotCar Dataset, and a resolution of 0.4332m/pixel for lidar localization against satellite imagery. The resolutions used correspond to a “zoom level” of 17 and 18 respectively when querying from Google Maps Platform, and are chosen based on the effective sensing range of radar and lidar.

**Fig. 14.**
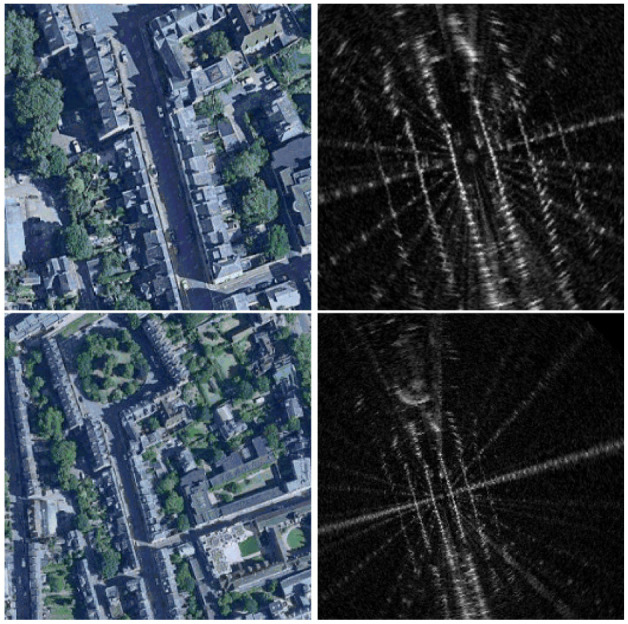
A pair of satellite and radar images queried using zoom levels of 18 (top) versus 17 (bottom) at roughly the same center position. Although “zooming in” can lead to a more refined resolution, certain regions far away are not seen in the resulting radar images, despite being observed by the sensor.

To study the effect of image resolution on solution quality, we created another dataset for radar localization against satellite imagery from the RobotCar Dataset, where the resolution is set to be 0.4332m/pixel, effectively “zooming in” on both the radar and satellite images. A comparison between images with the refined resolution and images used in the experiment in Section 6.1 is shown in Figure 14. We consider the same translation error in pixels for the initial estimate when comparing the two resolutions. The localization results are listed in Table 14.

**Table 14.** Mean error and error standard deviation for using refined resolution, evaluated for radar localization against satellite imagery. We also include results for a hybrid approach utilizing both resolutions..

	**Mean error (metric)**			**Mean error (pixel)**		**Error STD (metric)**		
	x(m)	y(m)	θ(°)	x	y	x(m)	y(m)	θ(°)
RobotCar, 0.8665m/pixel	3.44	5.40	**3.03**	**3.97**	6.23	4.44	7.50	**3.04**
RobotCar, 0.4332m/pixel	**2.23**	**2.42**	3.22	5.29	**5.74**	**2.49**	**3.02**	3.13
RobotCar, hybrid	2.56	3.60	3.03	5.91	8.32	3.78	5.45	3.04

Overall, choosing a resolution of 0.4332m/pixel resulted in slightly larger mean error in x and θ . Although zoomed-in images offer more refined resolution, without changing the size of the input images, by “zooming in,” measurements far from the sensor are essentially discarded, as shown in Figure 14, thereby limiting the amount of information being passed through the network.

Owing to the pixel-based nature of CNNs, the upper limit our method can handle in terms of initial translation offset is also inherently in pixels. Without changing the initial pose offset expressed in pixels, models trained with higher-resolution images offer a reduction in metric error, at the cost, however, of needing a smaller metric initial offset. In real-world applications, the coarse initial offset may be large, for example in places where direct GPS signals are occluded, and as such models trained with lower-resolution images are needed to handle such large initial offset. For a smaller metric initial offset, a model trained with higher-resolution images can be used to provide a more refined pose estimate.

With this in mind, we present a “hybrid” approach that utilizes both lower and higher resolution images. Specifically, given an initial offset in the range [−21.66m,21.66m] which corresponds to [−25,25] pixels with a resolution of 0.8665m/pixel, we first compute a solution using a model trained with lower-resolution images. Next, we take the estimated pose as the new initial translation offset and compute a refined solution using a model trained with higher-resolution images at 0.4332m/pixel. This “hybrid” approach allows for the best of both worlds: the lower-resolution model allows large metric initial offsets, whereas the higher-resolution model provides a further refinement, reducing the metric error. We demonstrate this approach for radar localization against satellite imagery on the RobotCar Dataset. The results are listed in the last row of Table 14: the metric error in both x and y are reduced compared with using a lower-resolution model only, by the sequential refinement with a higher-resolution model.

### 6.10. Choice of rotation increment

We have chosen a rotation increment of 2° when forming the rotation stack {B} for all of our experiments. Here we justify this choice by comparing the resulting rotation error for various increments.

By intuition, the solution error on θ should decrease with finer increments. However, a trade-off exists such that for the same range of initial heading error ( $\left[-\frac{\pi }{8},\frac{\pi }{8}\right]$ in all our experiments), the number of rotated images forming {B}, n, increases due to the smaller rotation increments. This, in turn, increases the memory usage during training. When training on two 1080 Ti GPUs, limited by the memory requirement, we could only afford a batch size of 16 when the increment is set to 1°, whereas we can use a batch size of at least 32 when the increment is 2° or larger.

As listed in Table 15, the mean error in θ when choosing 1° as the rotation increment is slightly larger than when using 2°, primarily because of the fact that the batch size is too small, in our hypothesis. We used a batch size of 32 for the experiments where the increment is 2°, 3°, and 4°, and it is clear that the solution error increases with larger increments and, thus, coarser sampling. For an increment of 4° we could also afford a batch size of 64, however the resulting θ error was higher than using 32 as the batch size.

**Table 15.** Mean θ error and error standard deviation for various rotation increments, evaluated for radar localization against satellite imagery on the RobotCar Dataset.

**Rotation increment**	**Mean error in θ(°) **	**Error STD (°) **
$1^{{}^{\circ}}$	3.29	3.91
$2^{{}^{\circ}}$	**3.03**	**3.04**
$3^{{}^{\circ}}$	4.04	4.93
$4^{{}^{\circ}}$	4.26	5.86

### 6.11. Handling larger initial offset

**Fig. 15.**
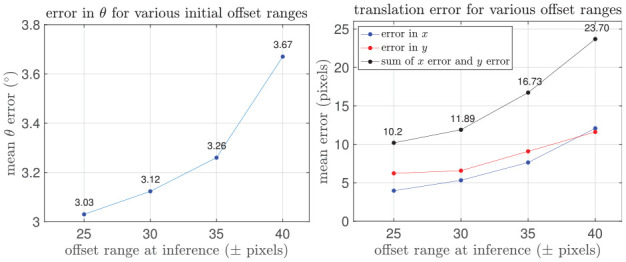
Mean error in rotation (left) and translation (right) with larger initial translation offset range, evaluated for radar localization against satellite imagery on the RobotCar Dataset.

So far, all experiments presented start from an initial translation offset of [−25,25] pixels, which corresponds to more than [−20m,20m] for the radar experiments in Section 6.1. In practice, the amount of offset our method can handle depends on the effective receptive field of the convolution layers in the encoder and decoder networks for image generation, namely $E_{a},E_{p}^{*},$ and D. If the offset is too large, the networks may not be able to encode and decode information needed to correctly generate ${\tilde{B}}_{\theta ,\alpha }.$ Supporting our intuition, Figure 15 shows that the solution error increases superlinearly with larger initial offset.

**Fig. 16.**
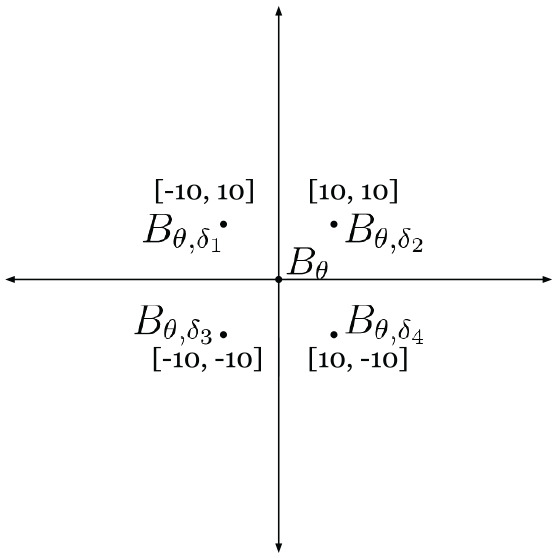
The center of image $B_{\theta }$ is shifted onto each of the four quadrants to produce four shifted versions.

Our method, however, naturally allows for a strategy to deal with larger initial offsets at inference time than in the training data, without the need to re-train. At inference, rather than using just $B_{\theta }$ during image generation, we can apply known translation offsets $\delta_{1},\delta_{2},\delta_{3},$ and $\delta_{4}$ to shift the center of $B_{\theta }$ onto each of the four quadrants. This is depicted in Figure 16, where as an example, we shift $B_{\theta }$ by [−10,10], [10,10], [−10,−10], and [10,−10] pixels to form $B_{\theta ,\delta_{1}},$ $B_{\theta ,\delta_{2}},$ $B_{\theta ,\delta_{3}},$ and $B_{\theta ,\delta_{4}},$ respectively.

**Fig. 17.**
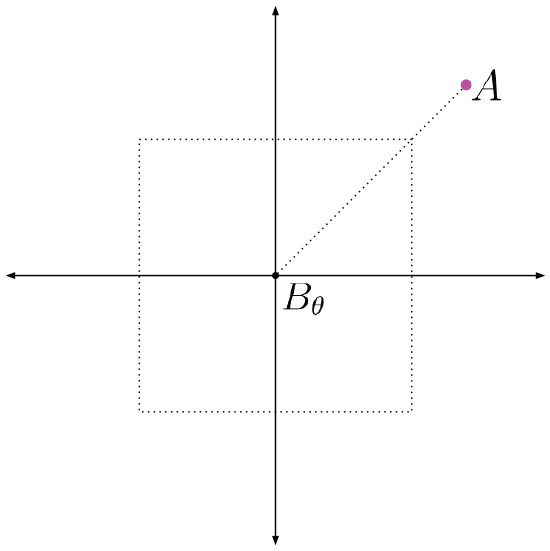
The unknown translation offset α between A and $B_{\theta }$ is larger than the networks are designed for.

**Fig. 18.**
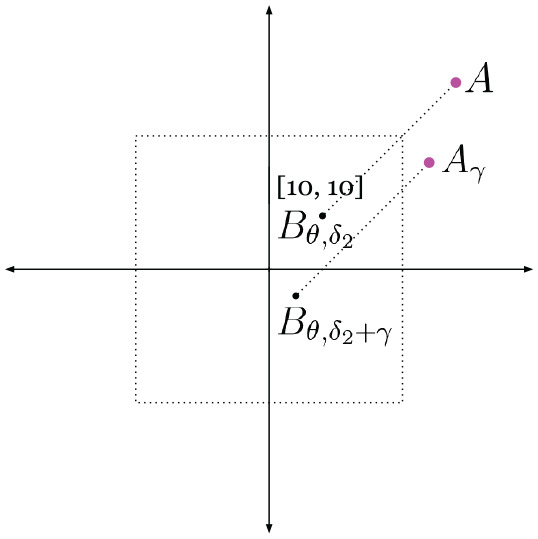
If we shift $B_{\theta }$ by [10,10] to form $B_{\theta ,\delta_{2}},$ then the offset between A and $B_{\theta ,\delta_{2}}$ is within what the networks are designed for. In this case, generating ${\tilde{B}}_{\theta ,\alpha }$ and ${\tilde{B}}_{\theta ,\alpha +\gamma }$ should both be accurate, as the offset in both cases are within what the networks are trained for.

Figure 17 depicts a case where the translation offset α between the satellite image A and $B_{\theta }$ is $\alpha ={\left[\begin{array}{cc} 35 & 35 \end{array}\right]}^{T}$ pixels. This is larger than the range of translation offsets in the training data, which is [−25,25] pixels, shown by the dashed box around the origin. However, the offset between $B_{\theta ,\delta_{2}}$ and A is ${\left[\begin{array}{cc} 25 & 25 \end{array}\right]}^{T}$ pixels, which is within the range our networks can handle, as shown in Figure 18.

Forming $E_{p}^{*}(A,B_{\theta })$ and $E_{a}(B_{\theta })$ during image generation might lead to incorrect results when generating ${\tilde{B}}_{\theta ,\alpha },$ as the offset between A and $B_{\theta }$ is larger than what $E_{p}^{*}$ is trained for. However, we can also generate ${\tilde{B}}_{\theta ,\alpha }$ using $E_{p}^{*}(A,B_{\theta ,\delta_{2}})$ and $E_{a}(B_{\theta ,\delta_{2}}):$

(22) $${\tilde{B}}_{\theta ,\alpha }=D(E_{a}(B_{\theta ,\delta_{2}}),E_{p}^{*}(A,B_{\theta ,\delta_{2}})).$$

It should be noted that such a combination does not suffer from the issue with large offsets, as discussed.

**Fig. 19.**
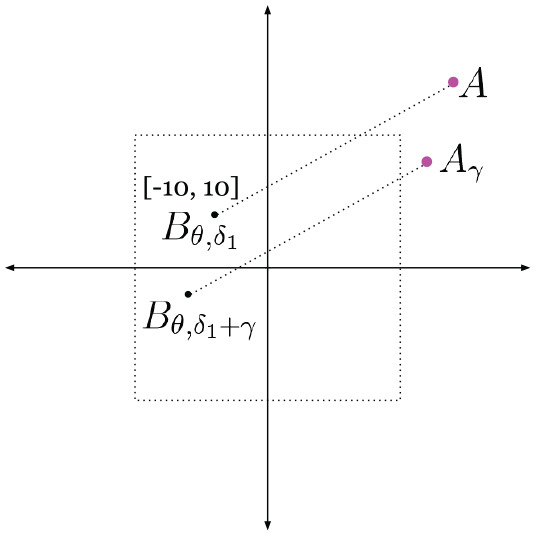
The resulting synthetic image will still be erroneous, if an incorrect quadrant is selected. Here the offset between $B_{\theta ,\delta_{1}}$ and A is larger than what the networks can handle. In this case, generating ${\tilde{B}}_{\theta ,\alpha }$ and ${\tilde{B}}_{\theta ,\alpha +\gamma }$ will both be problematic due to the issue with offsets.

The remaining question is then which shifted image from $B_{\theta ,\delta_{1}},$ $B_{\theta ,\delta_{2}},$ $B_{\theta ,\delta_{3}},$ and $B_{\theta ,\delta_{4}}$ to choose from. In Figure 19, as an example, we show that generating ${\tilde{B}}_{\theta ,\alpha }$ using $E_{p}^{*}(A,B_{\theta ,\delta_{1}})$ and $E_{a}(B_{\theta ,\delta_{1}})$ will also be problematic, as this combination also suffers from the issue with large offsets. The selection cannot be made ahead of the image generation as α is unknown. However, our method naturally allows for a strategy to select the correct quadrant to shift $B_{\theta }$ onto, without needing to know α a priori, using an introspective method similar in spirit to that presented in Section 6.4.1.

We can generate five versions of ${\tilde{B}}_{\theta ,\alpha }$ using $B_{\theta },$ $B_{\theta ,\delta_{1}},$ $B_{\theta ,\delta_{2}},$ $B_{\theta ,\delta_{3}},$ and $B_{\theta ,\delta_{4}},$ and introspect the quality of each ${\tilde{B}}_{\theta ,\alpha }.$ To do so, we apply a known shift γ to A to query for another image $A_{\gamma },$ and we can also shift each $B_{\theta ,\delta_{i}}$ by γ to form $B_{\theta ,\delta_{i}+\gamma }$ (or $B_{\theta ,\gamma }$ for $B_{\theta }$ ), as in Figures 18 and 19.

For each shift $\delta_{i}$ (and zero shift for $B_{\theta }$ ), we can take the combination $E_{p}^{*}(A_{\gamma },B_{\theta ,\delta_{i}+\gamma })$ and $E_{a}(B_{\theta ,\delta_{i}+\gamma })$ to generate ${\tilde{B}}_{\theta ,\alpha +\gamma },$ which should be pixel-wise aligned with $A_{\gamma }.$ If generating ${\tilde{B}}_{\theta ,\alpha }$ is problematic due to large offsets, then so will generating ${\tilde{B}}_{\theta ,\alpha +\gamma }$ be, as shown in Figure 19. On the other hand, if the networks can correctly produce ${\tilde{B}}_{\theta ,\alpha },$ they can also correctly produce ${\tilde{B}}_{\theta ,\alpha +\gamma },$ as shown in Figure 18.

For each shift $\delta_{i},$ we can compute a translation offset $\hat{\gamma }$ using ${\tilde{B}}_{\theta ,\alpha +\gamma }$ and ${\tilde{B}}_{\theta ,\alpha }:$

(23) $$\hat{\gamma }=\underset{p\in R^{2}}{\arg\;\max}{\tilde{B}}_{\theta ,\alpha +\gamma }^{†}★{\tilde{B}}_{\theta ,\alpha }^{†}$$

along with an error term $e=\|\hat{\gamma }-\gamma \|.$ For the five pairs of synthetic images, the one that results in the smallest e will be used and passed downstream to solve for $\hat{\alpha }.$ This forms an introspective approach for handling initial offsets larger than what the models are trained for, by augmenting the original input $B_{\theta }$ with various shifted versions.

Table 16 shows results on the RobotCar Dataset for radar localization against satellite imagery, where the initial offset is now in the range of [−35,35] pixels. We also include additional results where the networks are trained using the same offset range as at inference for comparison. Taking a model trained for an offset in the range [−25,25] pixels and evaluating directly with offsets in the range [−35,35] pixels, we resulted in higher errors compared with Section 6.1. However, with our input-augmentation approach by shifting $B_{\theta }$ and generating ${\tilde{B}}_{\theta ,\alpha }$ multiple times, we can handle larger initial offsets without sacrificing significantly on accuracy. This method, however, introduces additional computational cost as multiple forward passes of image generation are needed.

**Table 16.** Radar localization against satellite imagery evaluated on the test set of RobotCar, where the initial translation offset is large at inference. We also included results from Table 5 for reference..

**Offset range**	**Offset range**	**With input**	**Mean error (metric)**			**Mean error (pixel)**	
**at training (pixels)**	**at inference (pixels)**	**augmentation**	x(m)	y(m)	θ(°)	x	y
[−25,25]	[−25,25]	✗	3.44	5.40	3.03	3.97	6.23
[−25,25]	[−35,35]	✗	6.62	7.88	**3.26**	7.64	9.09
[−25,25]	[−35,35]	✓	4.67	**5.54**	**3.26**	5.39	**6.40**
[−35,35]	[−35,35]	✗	**4.28**	7.35	**3.26**	**4.94**	8.49

### 6.12. Modality transfer

An interesting application would be to train models using data for localization between one type of range sensor (e.g., radar) and satellite imagery, and evaluate for localization between another type of range sensor (e.g., lidar) and satellite imagery. This is particularly useful if we wish to evaluate using a specific type of range sensor at inference, but do not have the associated training data with approximately known trajectories to query for satellite images.

**Fig. 20.**
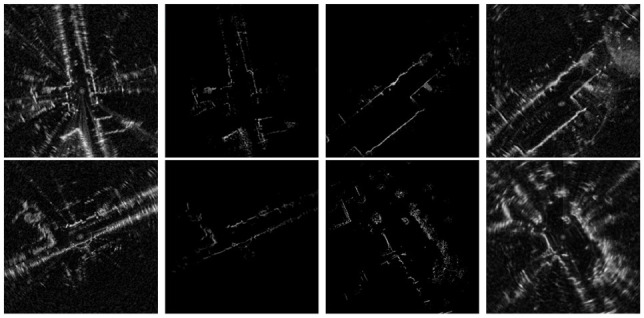
Qualitative results of CycleGAN for domain adaptation between a single scan of radar and lidar data. From left to right: a real radar image and its synthetic lidar image, a real lidar image and its synthetic radar image. Top: CycleGAN applied in Cartesian coordinate representation. Bottom: CycleGAN applied in polar coordinate representation.

Here we demonstrate how this can be achieved using unsupervised domain adaptation by image-to-image transfer. Specifically, we consider CycleGAN (Zhu et al., 2017) for transferring between radar scan images and lidar scan images. For transferring between images of single scans of radar and lidar data, we also implement the approach in Weston et al. (2020), where range sensor data are converted into polar coordinate representation prior to the domain adaptation. Figure 20 shows examples of radar and lidar images and their synthetic counterparts generated using our implementation of CycleGAN, for both polar and Cartesian coordinate representations.

At inference, given networks $E_{a},E_{p}^{*},D,H_{B},$ and $H_{\tilde{B}}$ trained for radar localization against satellite imagery, and a lidar image $B^{l},$ we can generate ${\tilde{B}}^{r}=f_{L\to R}(B^{l}),$ where ${\tilde{B}}^{r}$ is a synthetic radar image and $f_{L\to R}$ is a network that transforms a lidar image to a synthetic radar image trained using the objectives of CycleGAN (Zhu et al., 2017).

We can then use ${\tilde{B}}^{r}$ as the input for inferring rotation, conditional image generation, and learning pose estimation using the pipeline detailed in Section 4.

The same can be performed for the other way around, where given networks trained for lidar localization against satellite imagery, we wish to perform radar localization against satellite imagery at inference time.

Table 17 summarizes the results for modality transfer. Networks trained with one type of range sensor suffer from large localization errors when directly evaluated with another type of sensor. The errors are greatly reduced after applying domain adaptation and using transferred images as input. As shown in Figure 20, applying domain adaptation in polar coordinate representation led to more visually realistic synthetic images when transferring from lidar to radar data, and the smallest localization errors for networks trained on radar data and tested with lidar data. Applying domain adaptation in Cartesian coordinate representation, on the other hand, resulted in smaller errors for the reverse experiment. We use radar and lidar images of the same resolution in this experiment, and do not consider modality transfers that also involve a change in resolution.

**Table 17.** Localization results for experiments where the range sensor used is different between training and inference time, with and without using modality transfer, evaluated on the RobotCar Dataset. We also included results from Tables 6 and 14 for reference.

**Sensor in**	**Sensor at**	**With modality**	**Mean error (metric)**			**Mean error (pixel)**	
**training data**	**inference**	**transfer**	x(m)	y(m)	θ(°)	x	y
Lidar	Lidar	✗	1.54	1.85	2.29	3.55	4.27
Radar	Lidar	✗	6.39	8.64	4.71	14.74	19.95
Radar	Lidar	✓(Cartesian)	2.63	3.48	2.65	6.08	8.03
Radar	Lidar	✓(Polar)	**2.04**	**2.20**	**2.38**	**4.71**	**5.08**
Radar	Radar	✗	2.23	2.42	3.22	5.29	5.74
Lidar	Radar	✗	7.03	6.43	10.05	16.23	14.83
Lidar	Radar	✓(Cartesian)	2.74	**2.84**	**3.94**	6.32	**6.56**
Lidar	Radar	✓(Polar)	**2.73**	3.52	4.62	**6.31**	8.14

### 6.13. Further qualitative results

**Fig. 21.**
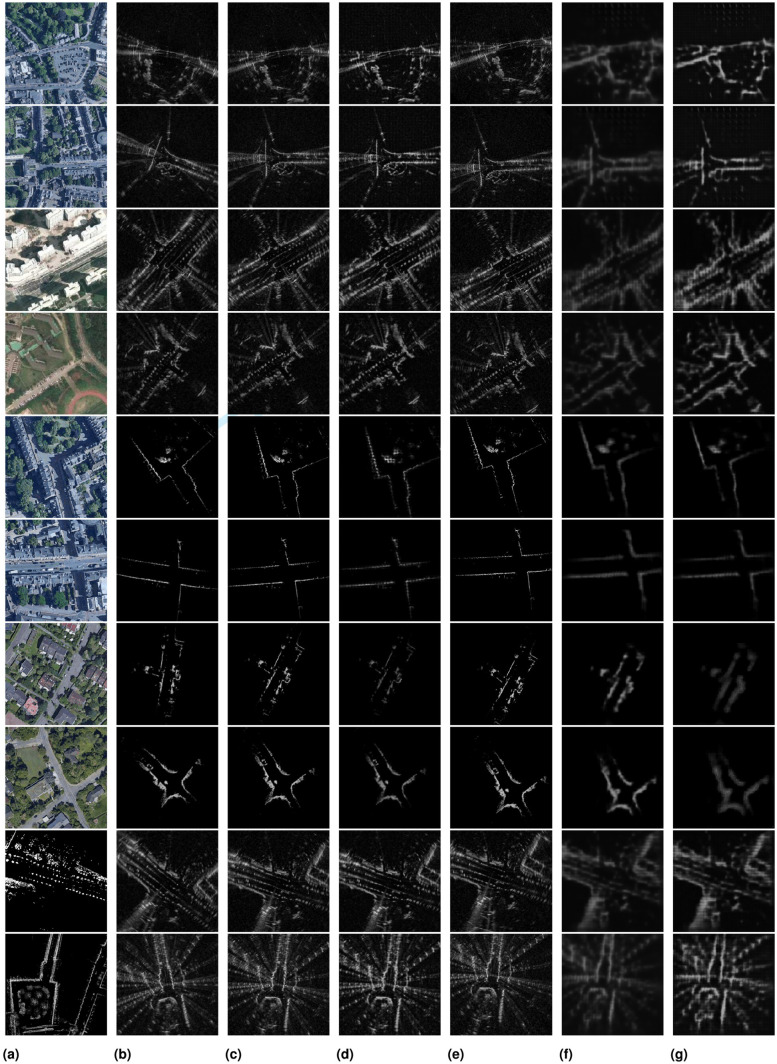
Images at various stages of our method: map image A (a), live data image B (b), output of rotation inference $B_{\theta }$ (c), embedding $B_{\theta }^{†}$ (d), pixel-wise aligned ground truth $B_{\theta ,\alpha }$ (e), synthetic image ${\tilde{B}}_{\theta ,\alpha }$ (f), and embedding ${\tilde{B}}_{\theta ,\alpha }^{†}$ (g). From top to bottom: radar localization against satellite imagery evaluated on RobotCar (rows 1–2) and MulRan (rows 3–4), lidar localization against satellite imagery evaluated on RobotCar (rows 5–6) and KITTI (rows 7–8), and radar localization against lidar map evaluated on MulRan (row 9) and RobotCar (row 10).

Additional qualitative results are presented in Figure 21 showing various stages of our methods for different modalities and datasets.

## 7. Conclusion and future work

We present self-supervised learning to address cross-modality, metric localization between satellite imagery and on-board range sensors, without metrically accurate ground truth for training. Our approach utilizes a multi-stage network that solves for the rotation and translation offsets separately through the generation of synthetic range sensor images as an intermediate step. Our method is validated across a large number of experiments for multiple modes of localization, with results on par with a prior supervised approach. A coarse initial pose estimate is needed for our method to compute metric localization. Therefore, a natural extension would then be to solve place recognition for a range sensor within a large satellite map as a prior step to metric localization.

## Notes

1. A mapping f:X→Y is *equivariant* to a group of transformations Φ, if for any ϕ∈Φ, there exists a transformation ψ∈Ψ such that ψ(f(χ))=f(ϕ(χ)),∀χ∈X.

2. See https://developers.google.com/maps/documentation/maps-static/intro/

3. See https://docs.microsoft.com/en-us/bingmaps/
